# Reward Maximization Justifies the Transition from Sensory Selection at Childhood to Sensory Integration at Adulthood

**DOI:** 10.1371/journal.pone.0103143

**Published:** 2014-07-24

**Authors:** Pedram Daee, Maryam S. Mirian, Majid Nili Ahmadabadi

**Affiliations:** 1 Cognitive Robotics Laboratory, Control and Intelligent Processing Center of Excellence, School of Electrical and Computer Engineering, College of Engineering, University of Tehran, Tehran, Iran; 2 School of Cognitive Sciences, Institute for Research in Fundamental Sciences (IPM), Tehran, Iran; VU University Amsterdam, Netherlands

## Abstract

In a multisensory task, human adults integrate information from different sensory modalities -behaviorally in an optimal Bayesian fashion- while children mostly rely on a single sensor modality for decision making. The reason behind this change of behavior over age and the process behind learning the required statistics for optimal integration are still unclear and have not been justified by the conventional Bayesian modeling. We propose an interactive multisensory learning framework without making any prior assumptions about the sensory models. In this framework, learning in every modality and in their joint space is done in parallel using a single-step reinforcement learning method. A simple statistical test on confidence intervals on the mean of reward distributions is used to select the most informative source of information among the individual modalities and the joint space. Analyses of the method and the simulation results on a multimodal localization task show that the learning system autonomously starts with sensory selection and gradually switches to sensory integration. This is because, relying more on modalities -i.e. selection- at early learning steps (childhood) is more rewarding than favoring decisions learned in the joint space since, smaller state-space in modalities results in faster learning in every individual modality. In contrast, after gaining sufficient experiences (adulthood), the quality of learning in the joint space matures while learning in modalities suffers from insufficient accuracy due to perceptual aliasing. It results in tighter confidence interval for the joint space and consequently causes a smooth shift from selection to integration. It suggests that sensory selection and integration are emergent behavior and both are outputs of a single reward maximization process; i.e. the transition is not a preprogrammed phenomenon.

## Introduction

To make an appropriate decision, our brain has to perceive the current state of the environment. However, even our best senses are noisy and can only provide an uncertain estimate of the underlying state. The biological solution for achieving the best perception is integration of uncertain individual estimates.

Human adults integrate sensory information, both across and within different modalities, with seemingly the purpose of reducing the uncertainty of their perception. The overwhelming majority of behavioral studies have shown that this uncertainty reduction happens in a statistically optimal fashion [1], [2]. One way to model this optimal integration is employing the Bayesian framework. In this framework and under some assumptions, the integration procedure is modeled by a weighted average of the individual sensors' estimates. Each sensor's weight is proportional to its relative reliability; i.e. inverse of its uncertainty. It can be shown that the reliability of the integrated estimate is higher than that of any individual's estimate.

Nevertheless, many behavioral studies indicate that this optimal behavior, and in some cases even its neural foundations, are not present at birth. Furthermore, it is only in the later stages of development that multisensory functions appear and take the main role in multisensory decision makings; see [3] for a comprehensive review. An increasing number of studies in different sensory modalities on adults and children have shown that, unlike adults, children make their judgments based only on one of the available sources of information. Some instances of this sensory selection behavior have been observed in visual and haptic modalities for size and orientation discrimination [4], visual landmarks and self-motion information for navigation [5], and visual stereoscopic and texture information for estimating surface slant [6].

The interesting open questions here are “Why does optimal integration occur so late?” [7], why there is a tendency in sensory selection in children, and finally, how and based on what measures does the transition from sensory selection at childhood to sensory integration at adulthood happen. While there are a considerable number of hypotheses regarding the reasons behind these phenomena (see [6], [3], [7]), to our knowledge, no existing study has addressed these three questions with a unified computational model. The primary aim of this research is to investigate the computational advantages of the transition from sensory selection at early ages toward multisensory integration at adulthood. The second goal is to check if the above three questions can be addressed by a single computational model.

We hypothesize that this selection and integration are emergent behavior of a single reward maximization system. To verify our hypothesis, we propose a mathematically sound and general reward dependent learning framework (see Method) and test it in a multisensory localization task (see Experiments and Results). The learning method is value-based [8] [9] and progress of learning in the framework corresponds to development of the agent over age. This choice is natural as there are supporting studies indicating that the multisensory integration is not innate and there should be a learning mechanism behind its development (see [3], [10]). Furthermore, this framework does not require most of the strict mathematical assumptions that are building blocks of the conventional Bayesian framework, which are widely used to explain multisensory integration.

## Method

Consider an agent with *k* sensors 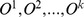, where 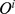 is the observation space of the 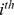 sensor. Furthermore, assume that the environment is fully observable in the Cartesian product of the observation spaces, i.e. 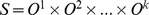. At each time step, the agent should choose an action from its action set 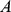 according to the perceptual input (state) 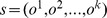, where 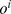 is the current reading of the 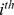 sensor. After performing the action, the agent receives an immediate reinforcement signal (reward) 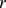 from the environment. It is assumed that all the reward distributions, corresponding to the state-action pairs, are unknown with support in 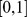. The goal of the agent is to maximize the total amount of reward it receives over its lifetime. To achieve this goal, the agent should learn the appropriate action in response to members of the joint sensory space 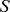.

**Figure 1:**
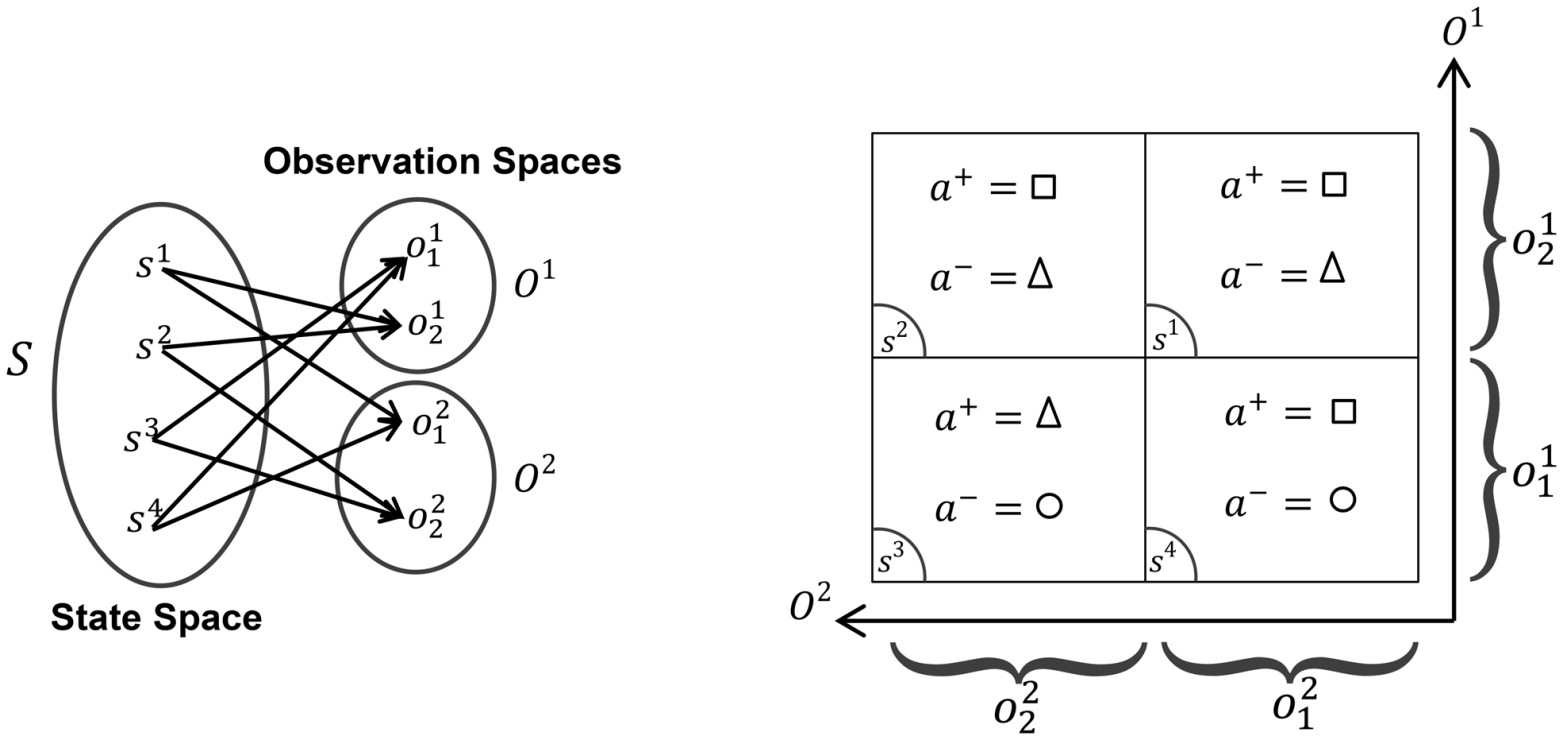
Different types of perceptual aliasing in subspaces. 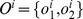
 represents the observation set of the *i^th^* sensor for i = 1, 2. 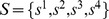 is the state set and *A = *{○,□,Δ} is the action set of the agent. 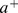 and 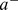 are the best and the worst actions in the given state, respectively. Accumulated experience in 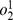 is a perfect generalization for 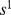 and 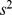, since these two states have the same optimal policy and 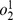 is common between them. In contrast, accumulated experience in 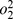 is garbage information because functionally different states are mapped to the same observation. The situation for 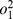 and 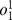 is a little different. Only for the best action in 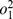 and the worst action in 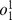 we have the generalization, however, for the other action this is not the case.

The primary challenge here is that the state space 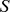 is high dimensional. Therefore, to learn the best action corresponding to each member of 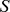, a large number of experiences (samples) is needed. This problem is known as the curse of dimensionality. One way to tackle this problem is to use the experiences in the subspaces of 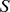
”, such as *O^i^*, for decision making [11], [12]. However, the environment in the eyes of *O^i^* is partially observable, which creates a many-to-one mapping between real states of the environment and observations in *O^i^*. This problem is known as Perceptual Aliasing (PA) [13] and is avoided in general. Nevertheless, PA might be beneficiary in learning a task [11], since it can partially free the learner from the curse of dimensionality if states sharing the same *o^i^* have similar optimal policies. PA might be helpful at the early stages of learning as well, where learning a moderately rewarding policy over *O^i^* is faster than learning a policy with the same reward over the joint space *S*. In these two cases, learning in the subspaces results in generalization of experiences. In contrast, PA can be very undesirable when functionally different states of the environment, i.e. states with very different policies, are mapped to a same observation in *O^i^*. This case of PA turns the accumulated experience in that subspace into “garbage” [14]. Figure 1 illustrates these concepts in a simple example. Our proposed statistical test (see Generalization Test) has the ability to detect different cases of perceptual aliasing that are illustrated in the figure.

**Figure 2:**
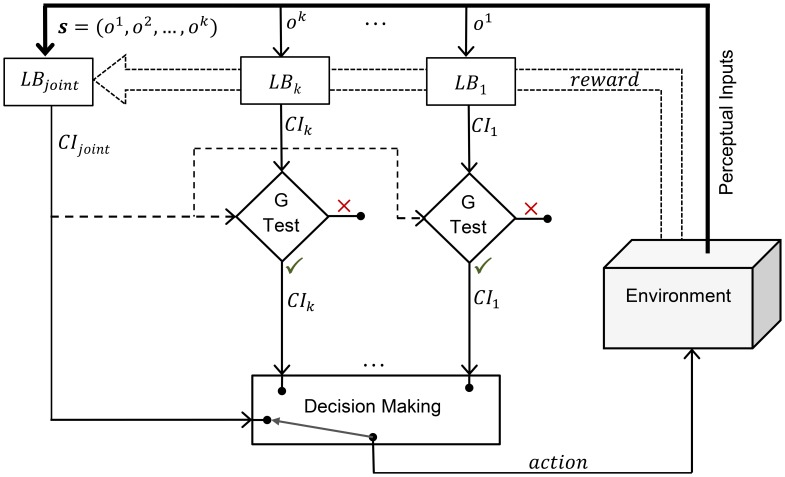
A schematic overview of the proposed framework for multisensory learning and decision making. *s = (o^1^,o^2^,…,o^k^)* is the perceptual input, 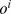 is the current reading of the *i^th^* sensor, and 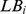 is the learning block of the *i^th^* sensor. For each action and based on the previously received rewards, each learning block calculates a confidence interval (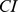) on the mean of the reward distribution corresponding to the given observation and action pair. The proposed Generalization Test (G Test), tests the generalization ability of the individual source against the joint space. In case that an individual source passes the G Test, its confidence interval will be considered in the decision making phase. In decision making phase, an appropriate action based on the given intervals will be selected which considers the exploration and exploitation trade-off.

In order to benefit from PA and to avoid its harms, a statistical test is proposed to discriminate estimates of the expected reward which are instances of generalization (beneficial cases of PA) from garbage information. The proposed test is in part inspired from McCallum's work on learning with incomplete perception [15]. Then, a selection policy for choosing the most reliable source of information is employed. Finally, according to the selected information, a decision making policy has been introduced which considers the exploration and exploitation trade-off. A schematic overview of the proposed method, including the Generalization Test (G Test) and the Decision Making phase, is illustrated in Figure 2. In the following subsections, the proposed multisensory learning and decision making method is explained in detail.

In general, there are two approaches for learning a task, learning through labeled samples and learning by interaction. State estimation in a supervised setting requires having the specifications of the states at hand. Nevertheless, in reality we should learn the states either directly or through learning the optimal policy. In the problem at hand, the agent begins its life in a tabula rasa state and there is no information available regarding the observation models of sensors and the relation between the agent's sensory space 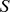 and its action space 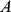. Furthermore, the only teacher that the agent can interact with is the environment. Therefore, only through interactions with the environment, the agent can learn to act properly. In this problem we are not interested in learning the observation models of individual sensors nor do we have the necessary sources of feedback to do this. Therefore, this problem is different from the conventional supervised learning where a teacher provides a set of labeled data, and the agent needs only to learn the observation models of sensors and perform a state estimation task.

### 1. Modeling

The actual value of choosing action 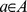 when the agent is in state *s*  =  (*o^1^, o^2^,…,o^k^*) is denoted as 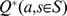, and its estimated value as 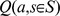. All the estimated values (Q-values) are represented in a 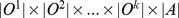 dimensional table, known as Q-table. Q-values are updated after each time step using 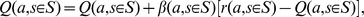
where 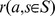 is the reward received after performing 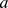 in 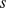, and 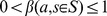 is the learning rate for the given state and action. We assume that the reward distributions are fixed throughout the learning; i.e. the environment is stationary. In stationary environments, it is rational to employ 
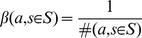
, where 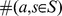 is the sample size, i.e. the number of times that action *a* is performed in state *s*. By using this learning rate, the above equation becomes identical to the incremental update formula for computing the average reward [8]. Therefore, Q-values are the sample means and 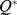
s are the actual means of the underlying reward distributions.

As it will be explained in the following sections, we need confidence intervals on 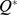 s for our generalization test and decision making method. For a moderately large number of samples, we can create a confidence interval on 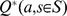 using the following bound [16]:
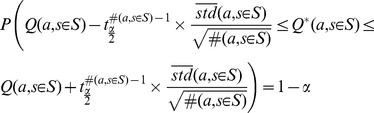
(1)


In (1) 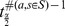 is the Student t distribution with 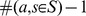 degrees of freedom. The parameter 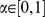 controls the confidence that 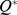 will fall inside the confidence interval. Finally, the value 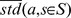 is the estimated standard deviation of the underlying reward distribution defined by
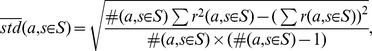
where 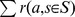 is the sum of the rewards and 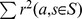 is the sum of the squares of the rewards received by performing 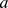 in *s*.

The confidence interval in (1) is mathematically valid when either the number of samples (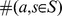) is moderately large or when the reward distribution is Normal (Gaussian). Although these conditions may seem rather restricting, in our experience, bound (1) works reasonably well in most practical cases.

When the sample size is not sufficiently large or the reward distribution is not Gaussian, we may use Chebyshev's inequality to calculate the confidence interval. To do so, we need the true standard deviation of the reward distribution, which is not available in general. However, defining the reward distribution in the interval 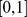, the maximum possible value for the variance is 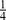. Then a very conservative Chebyshev's inequality is
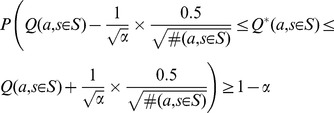
(2)


Although bounds (1) and (2) are similar in essence, bound (2) is very conservative but independent of the reward distribution. Conservativeness of (2) has roots in not taking into account the type of the reward distribution and its estimated variance. This lack of prior assumptions will result in extremely conservative intervals in cases that the variances are very small or even zero. In situations like these, it is better to employ the “variance-aware” inequality proposed in [17]:
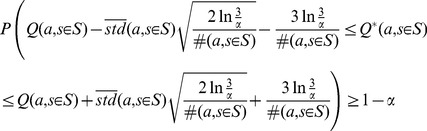
(3)


In this study, we are mainly interested in the *length* of the confidence intervals and their *relative length* to each other. Generally, by visiting new samples, the length of all the intervals in bounds (1), (2), and (3) diminishes gradually. Therefore, as we will see in the following sections, all the mentioned intervals are applicable in our algorithm. In Discussions and Conclusions section, a discussion on a number of practical points concerning these bounds is provided.

For individual sensors, 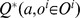 denotes the actual mean and 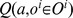 denotes the sample mean of reward, received by performing action 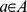 when the *i^th^* sensor's observation is 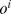. We can create a confidence interval on 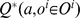 by using the same procedure and only replacing the following variables in bounds (1), (2), or (3):
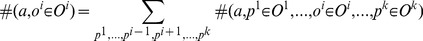
(4)

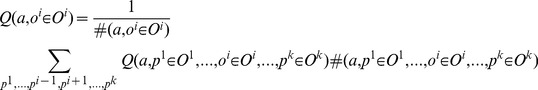
(5)


The above equations express the marginal values for the *i^th^* sensor.

In order to calculate 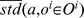 we also need to calculate two more terms:
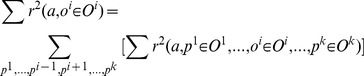
(6)

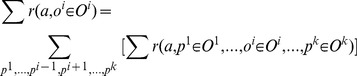
(7)


Calculation of (4)–(7) does not need extra learning trials because, these variables are calculated by marginalization of statistics of the joint space 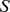.

### 2. Generalization Test

A statistical test is proposed to answer the following question:

*Is perceptual aliasing in 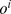, a beneficial case of generalization for action 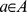,or a harmful case of “garbage” information?*

Based on our modeling, we can restate the question as “is 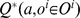 a reasonable representation of 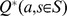
?”, where 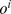 is the current observation of the *i^th^* sensor and *s  =  (o^1^,o^2^,…,o^k^)*. However, as previously mentioned, 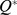 s are unknown. As such, we use their confidence intervals by employing either bounds (1), (2), or (3). We denote the confidence interval on 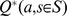 as 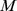 and confidence interval on 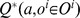 as 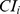.

To validate the generalization ability of 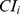, we need to test whether 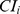 and 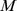 are estimating the same value (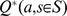). However, due to perceptual aliasing (many-to-one mapping), 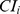 has also experienced all the rewards used in the calculation of 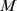. Hence, checking the significance of their difference does not provide useful information. The proposed idea here is to extract the common experiences between 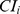 and 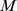, and then perform a statistical test on the residuals of 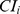, and 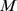. The procedure of extracting common experiences from 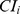 is as follows:
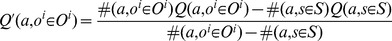
(8)

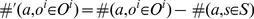
(9)

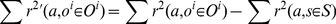
(10)

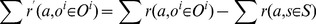
(11)


By using the variables on the left side of the above equations, a new confidence interval 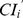 can be created using any of bounds (1), (2), or (3). For each action, 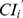 represents the intervallic estimate of the mean of a reward distribution created from experiences in the current observation of the *i^th^* sensor, minus the experiences in the current state of the environment. If there exists an intersection between 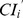 and 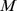, then there is a good chance that 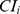 and 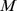 are estimating the similar expected value of rewards (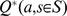). In other words, it means that the perceptual aliasing in 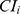 is a case of generalization. The proposed test states that at each time step for action 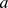:
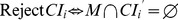
(12)


Based on (12), we can expect the following behavior in different stages of learning:

- During initial steps of learning (when sample size is very small), 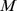 and 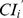 both have large confidence intervals. Consequently, 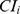 will be able to pass the proposed test in most time steps. Due to the low uncertainty in 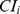, this behavior is desirable during initial steps.
- By gaining new samples, both 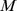 and 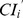 shrink. Therefore, the 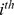 sensor will be able to pass the test only if its experiences are a good generalization of 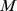
's experience.
- As the sample size for 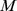 increases, its interval becomes smaller and smaller to a degree where it dwindles to only contain 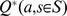. The same thing happens for 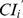 but it will converge to a different point. As a result, the test will reject all the individual sensors.

### 3. Decision Policy

As mentioned earlier, the agent starts with no prior information about the environment and the task at hand. Consequently, throughout the learning it faces the dilemma of gaining new experience by choosing one of the less explored decisions or exploiting the past experiences by selecting one of the well-rewarded decisions. This problem is known as the exploration versus exploitation trade-off [8].

At each state 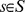, it can be assumed that there are 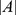 unknown reward distributions which correspond to each action in the action set 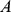. The best action 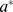 is the one corresponding to the distribution with the greatest mean, i.e. 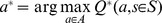. However, 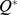 s are unknown and the agent should make the decision based on their estimates. A good decision policy should consider both the Q-value (sample mean statistic) and the uncertainty regarding its expected value. The value of the sample mean controls the exploitative selections, while its uncertainty controls the explorative decisions. Clearly, the uncertainty of the sample mean tends to zero as the number of samples tends to infinity, resulting in a smooth transition from exploration to exploitation as the number of samples increases.

A well-studied family of decision policies, which considers these two criteria, works based on the idea of creating an upper confidence interval on the mean of each reward distribution. Based on the calculated upper bounds, the decision policy selects the action with the greatest upper confidence interval [18]. This idea is known as “optimism in face of uncertainty principle.” It has been proved that variations of these decision policies, such as UCB1 [19], achieve logarithmic expected regret, i.e. the expected loss due to the fact that the agent does not always choose the optimal action, uniformly over the total number of samples of the given state. This amount of regret is the smallest possible expected regret, up to a constant factor. Fortunately, in the proposed approach we have already employed confidence intervals on the means of the reward distributions. The only difference in our problem is that we have a set of confidence intervals, instead of one, for each action. Therefore, we need to integrate available confidence intervals to one, and then employ the mentioned idea.

One can devise various methods for integrating a set of intervals. However, in this study we are interested in finding, specifically, the source of information that has the greatest impact on the final decision. As a result, we reduce the integration problem to selection of one of the available intervals as the representative interval for the given action. We propose two methods for this interval selection. The first method works by the idea of selecting the Most Optimistic Source (MOS), while the second method chooses the Least Uncertain Source (LUS). Details of these methods are as follows:

At each state 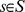 and for each action 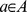, given a set of confidence intervals of individual sensors which were able to pass the previously mentioned test (12), the MOS method selects the interval with the greatest upper bound. The LUS method, on the other hand, selects the interval with the shortest length. The upper bound value of the selected interval will be used as the representative value for action 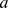. However, if this value is greater than 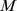
's upper bound, then 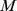
's upper bound will be used as the representative value. The reason behind this constraint is that, regardless of its great uncertainty, 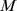 is still the most reliable (with lowest aliasing) source of information regarding the actual mean of the underlying reward distribution. Therefore, any value greater than 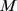
's upper bound is unrealistically optimistic. The idea behind LUS is that shorter intervals indicate lower uncertainty, and it is always desirable to attend the least uncertain source of information for decision making. The pseudo-codes of the MOS and LUS methods are shown in Table 1 and Table 2. For bound 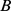, the notations 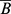 and 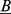 represent the upper bound and lower bound values of 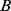, respectively.

**Table 1:** The function that implements MOS method.

function MOS(*M, Accepted*)	
Input: *_M_* is the confidence interval on the joint space, *Accepted* is the array storing confidence intervals on the sources that passed the generalization test	
1:	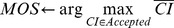
2:	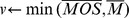
3:	**return** *v*

**Table 2:** The function that implements LUS method.

function LUS(*M, Accepted*)	
Input: *_M_* is the confidence interval on the joint space, *Accepted* is the array storing confidence intervals on the sources that passed the generalization test	
1:	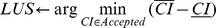
2:	**if ** 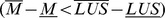 ** then ** 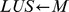
3:	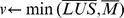
4:	**return** *v*

After choosing an upper bound value (with either MOS or LUS methods) for all the actions, the action with the maximum upper bound value is selected as the final decision. By performing the selected action, the environment returns the reward 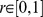. The complete pseudo-code of the proposed method is shown in Table 3. The only parameter that needs to be initialized is 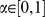, where 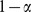 is the confidence coefficient of confidence intervals.

**Table 3:** The proposed Algorithm for Multisensory Learning and Decision Making.

Initialize 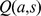 , 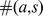 , 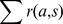 , **and** 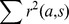 **to zero** 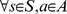				
1:	**Repeat** at each time step			
2:		*s = (o^1^,o^2^,…,o^k^)*		
3:		**for each** 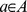 **do**		
4:			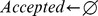	
5:			**for each** sensor 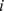 **do**	
6:				Calculate 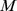 , 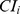 , and 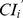 based on either bounds (1), (2), or (3)
7:				**if 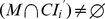 then 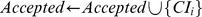 **
8:			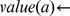 MOS( 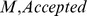 ) or LUS( 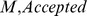 )	
9:		Perform 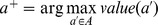 , observe reward 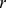		
10:		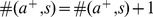		
11:		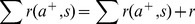		
12:		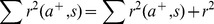		
13:		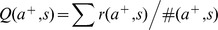		
14:	**Until** the end of the learning			

## Experiments and Results

**Figure 3:**
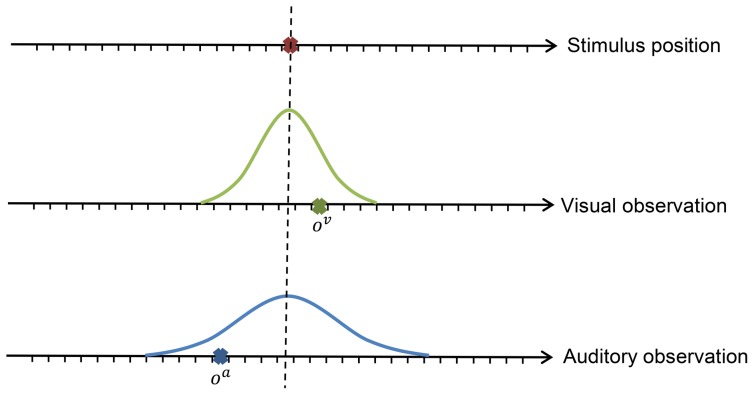
Stimulus and observations by the auditory (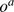) and the visual (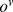) sensors. Observations are based on Gaussian noise models. Variances control the reliability of each sensor.

The task is a modified version of the localization task in the visual and auditory modalities [2] [20]. The simulation setup is based partly on [10]. At each time step, a stimulus is generated randomly in one of the 30 discrete positions and each sensor observes a noisy representation of it. The observation noise for each sensor is modeled by a Gaussian distribution with standard deviation 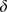; see Figure 3. After observing the stimulus through its sensors, the agent chooses one of the 30 discrete positions as the desirable action and receives an immediate reinforcement value in 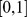:
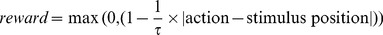
(13)


We used 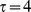, which indicates that only actions (estimates) within a radius of three units from the stimulus position receive positive rewards.

The agent has no prior information about the task, the observation models, and the relation between the sensory space and actions. Therefore, throughout the learning, it should learn the appropriate action only based on the sensory inputs and previously received rewards. On the other hand, the optimal Bayesian observer [2] assumes that all of the mentioned information is available and chooses its action according to the following integration rule:
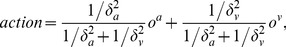
(14)where 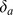 and 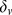 are the standard deviations of the Gaussian noise models for the auditory and visual inputs, respectively. Moreover, 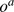 and 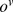 are the representations of the stimulus in the auditory and visual observation spaces. Behavioral studies have shown that adults integrate information from sensors in a statistically optimal manner which based on the Gaussian observation models, can be formulated by equation (14).

In all the following experiments, the proposed method uses the Cartesian product of the observation spaces of all the sensors for its state space. The agent's learning and decision making is based on Table 3.

### Experiment 1

In the first experiment we use 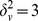 and 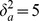 (see Figure 3). In order to validate our method, we employ three different agents. Two of the agents (Visual and Auditory agents) use only the individual sensors which will result in a state-action space of size 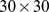 for each. The third one (Visual
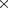
Auditory agent) uses both sensors for its learning and decision makings and has a state-action space of size 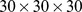. For these three agents, we employ the UCB1 policy [19] for decision making. UCB1 calculates upper bounds on the means of the reward distributions based on the Hoeffding inequality. At each state 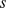, UCB1 chooses the action that maximizes
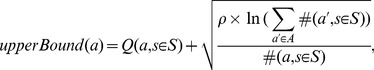
(15)where 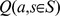 is the average reward obtained from performing action 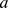 in state 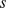, 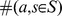 is the number of times 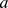 has been selected in 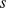, and 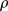 is the exploration coefficient [17]. In the original version of UCB1, 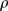 is set to 2. However, this value results in a high exploration rate. We use 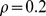 in all the experiments to increase the speed of learning for the rival agents.

It should be noted that when we use initial capital for a sensor, we are referring to the agent that learns in that sensor space. For instance, Visual refers to the agent that uses only the visual sate space for its learning.

**Figure 4:**
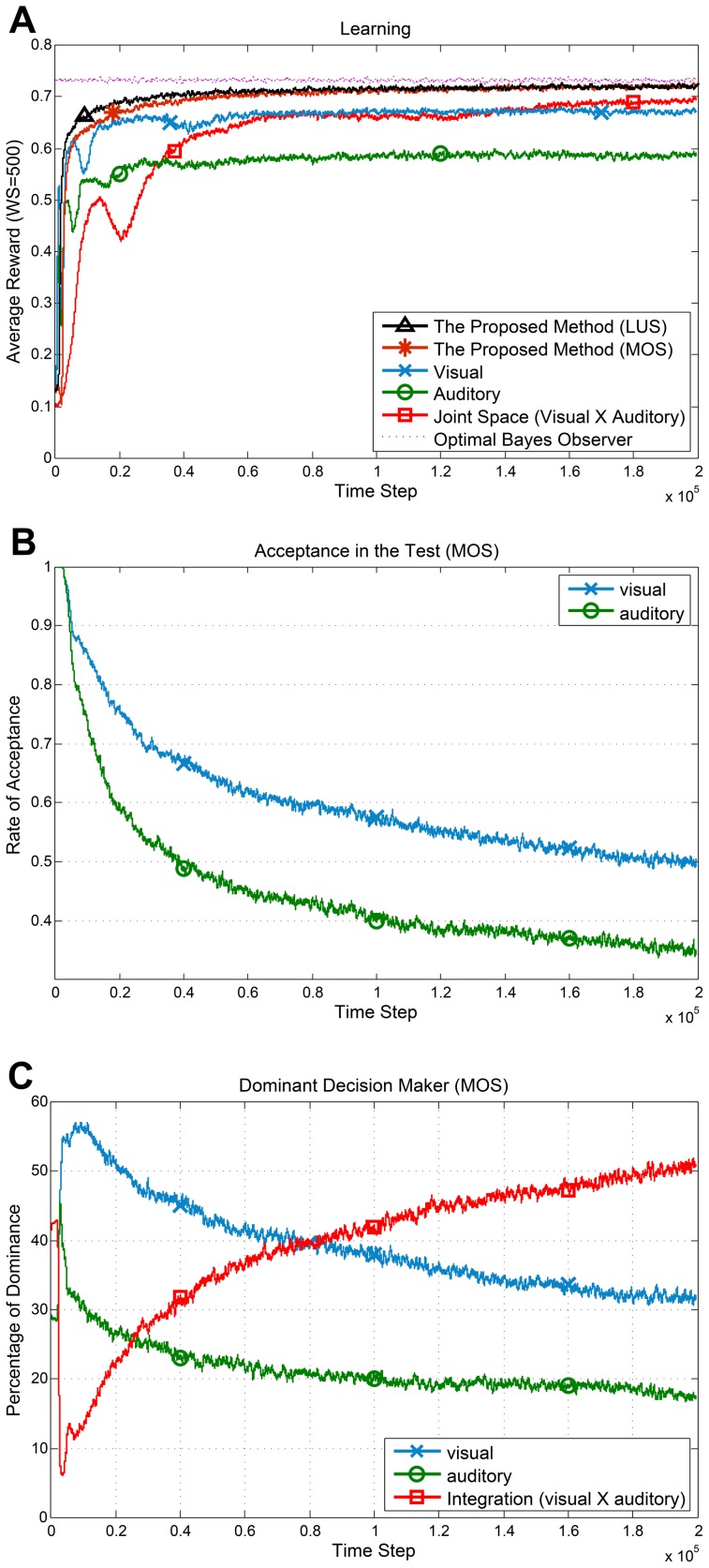
Performance and behavior of the method in the localization task. All graphs are results of averaging over 20 independent runs and passing a moving average window with size 500. (**A**) Average reward for all agents. For the proposed methods (MOS and LUS), we used Table 3, employing bound (1) with 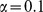 for calculating confidence intervals. The rival methods employ the UCB1 policy on the individual sensors and on the joint space. (**B**) Average acceptance rate (1–rejection rate) of the individual sensors in the proposed method (MOS). (**C**) The average dominancy percentage of each source in decision making (MOS). In the first half of learning steps, vision is the dominant sensor while the agent prefers the integrated sensory data in the rest of learning steps.

The average reward against the time step for all the agents and the optimal Bayesian observer are shown in Figure 4A. For the proposed methods (MOS and LUS), we employed bound (1) with 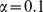. As can be seen in the figure, the proposed methods have a noticeably faster learning and higher rewards compared to the Visual
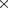
Auditory agent. The Visual and the Auditory agents both have a smaller state space (only one sensor) which results in a fast learning during initial time steps. However, due to their partial perception, they can never reach the performance of the optimal Bayesian observer.

To evaluate the proposed generalization test (see Figure 2 and Generalization Test) for the proposed method (MOS), the average outcome of the test for the chosen action against the time step is shown in Figure 4B. The value in the vertical axis specifies the rate of acceptance in the test which is 1–rejection rate. The test completely accepts the individual sensors during initial steps. This is in line with having a generalization power in the individual sensors due to more samples. Nevertheless, as the joint space learning improves, the rate of acceptance for the individual sensors decreases. This is because of sufficient experience accumulation in the joint space and existence of perceptual aliasing in the individual sensor spaces. This decline is more noticeable for the auditory sensor which is less reliable.

To investigate the decision making behavior of the proposed method (MOS), the average dominancy percentage of each source of information over time is shown in Figure 4C. In the initial steps of learning, vision is the dominant modality. However, as the time step increases there is a tendency to rely on the joint space for decision making (sensory integration). Considering Figure 4A and Figure 4C we can conclude that as the average reward received in the joint space increases, the proposed method gradually switches its decision policy from selection to integration. This behavior is comparable to the humans' shift from sensory selection at childhood to sensory integration at adulthood.

Performance criteria for different variations of the proposed method and the Visual
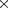
Auditory agent are illustrated in Table 4.

**Table 4:** Analyzing the learning speed and the behavior of different methods for Experiment 1 and 2.

Percentage of accumulated reward Learning Method		Experiment 1				Experiment 2				
		# time step	Percentage of dominance			# time step	Percentage of dominance			
			V	A	I		V	A	N	I
60%	Joint Space	38,113	0	0	100	1,141,640	0	0	0	100
	MOS, bound(1), 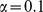	8,200	56	32	12	12,455	62	27	7	4
	LUS, bound (1), 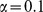	5,010	56	32	12	5,557	61	32	5	2
	MOS, bound (2), 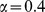	7,901	62	37	1	10,828	60	32	8	0
	LUS, bound (2), 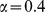	5,599	64	35	1	8,852	60	29	11	0
75%	Joint Space	81,179	0	0	100	2,437,811	0	0	0	100
	MOS, bound (1), 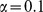	17,393	52	28	20	33,911	62	25	4	9
	LUS, bound (1), 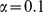	10,341	58	29	13	17,289	57	31	5	7
	MOS, bound (2), 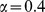	17,854	61	37	2	35,979	67	28	4	1
	LUS, bound (2), 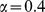	14,138	67	31	2	35,461	68	27	4	1
90%	Joint Space	348,945	0	0	100	10,036,225	0	0	0	100
	MOS, bound (1), 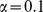	72,689	40	20	40	1,148,066	43	20	2	35
	LUS, bound (1), 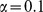	43,281	50	25	25	974,986	39	21	4	36
	MOS, bound (2), 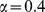	96,437	53	38	9	1,767,754	58	30	3	9
	LUS, bound (2), 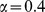	94,204	66	25	9	1,831,145	61	24	3	12

The performance criterion is the number of time steps needed to reach a certain percentage of the Bayesian optimal observer's accumulated reward. V = visual, A = auditory, N = noise, I = integration.

In Figure 4A there is a temporary decline in the average reward of the individual sensors and the joint space agents. The reason behind these declines is the inherent temporary exploration in UCB1. In UCB1, the policy calculates 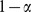 upper confidence bound where 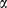 has an inverse relation with the total number of samples in state 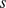 (the logarithmic term in equation (15)). Therefore, if an action has not been visited in a state for a long time, this term forces the agent to choose that action. For large state-action spaces, it creates temporary exploration phases in the learning. This exploration is beneficial in non-stationary environments, however, our environment is stationary and the exploration results in the observed decline. We reduced the exploration effect by using small 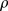 in (15). We tested the individual sensors and the joint space agents using constant alpha and different types of confidence intervals as well and the significant superiority of the proposed method was still intact.

#### A non-stationary change in the environment

**Figure 5:**
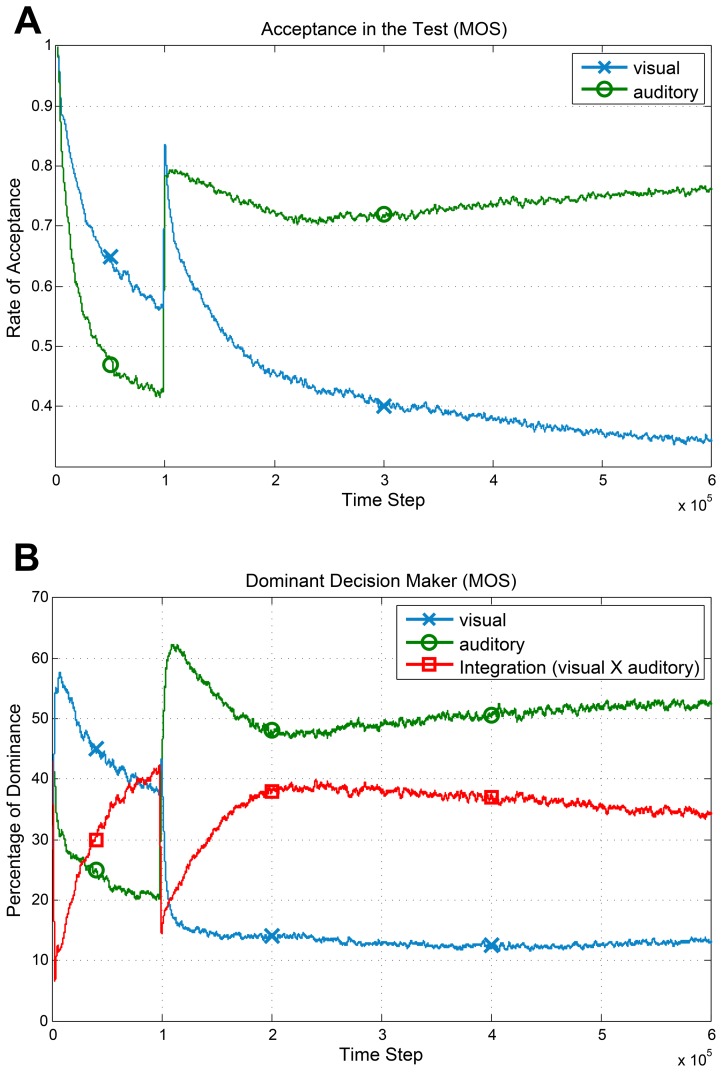
Performance of the method (MOS) in response to an unexpected change in the environment. At time step 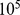 the visual sensor fails and its variance changes to the highest possible value. All graphs are results of averaging over 10 independent runs and passing a moving average window with size 500. (**A**) Average acceptance rate (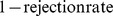) of the individual sensors. (**B**) The average dominancy percentage of each source in decision making (MOS). After failure of the visual sensor, the method detects this change and relies on the auditory sensor for decision making.

Having a stationary environment is one of the basic assumptions we made. To investigate the effect of an unexpected change in the environment, we decreased the reliability of visual sensor to the lowest possible value at step 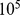. The underlying reward distributions for the visual sensor and the joint space changed accordingly. As Figure 5A shows, this change is detected by the proposed test. As a result, the rate of acceptance of the visual sensor noticeably decreases after step 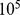. However, in the decision making section, only the MOS method could cope with this disturbance and the LUS method failed to adapt its behavior; as it relies more on the joint space. The percentage of dominance for each source of information in the MOS method is shown in Figure 5B. After time step 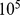, the agent relies more on the auditory sensor and only about 13% of decisions are made according to the visual data. We will discuss more on non-stationary environments in Discussions and Conclusions.

#### Parameter setting

**Figure 6:**
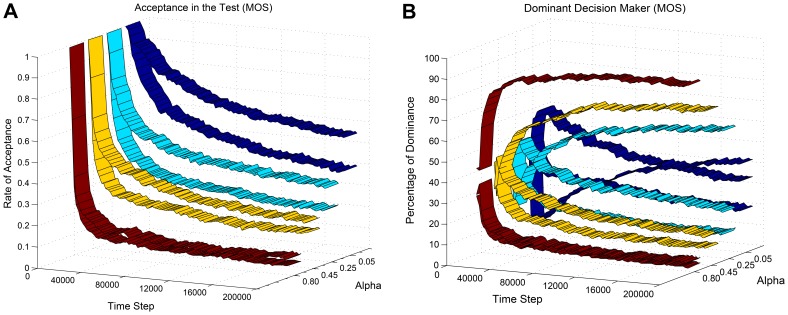
Impact of 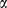. We used four different values (0.05, 0.25, 0.45, 0.80) for 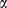 from being conservative to liberal in terms of confidence. All graphs are results of averaging over 10 independent runs and passing a moving average window with size 500. (**A**) Average acceptance rate (1–rejection rate) of the individual sensors in the proposed method (MOS). The upper/lower ribbon for each value of 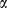 represents visual/auditory sensor. By increasing 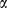, the test becomes harder for the individual sensors to pass. (**B**) The average dominancy percentage of each source in decision making (MOS). For each value of 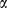, the ascending ribbon represents integration and the two descending ribbons represent selection of visual and auditory sensors. Increasing 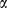 results in earlier cross of the ascending and the descending ribbons; i.e. earlier switch from selection to integration.

The method (Table 3) does not need any tuning and the only open parameter is 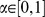, initialized at the beginning of the learning. Alpha defines the agent's characteristic; smaller value for 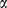 results in larger confidence intervals which means more tendency toward exploration than exploitation. Moreover, small value for alpha makes the test easier for individual sensors to pass, and as a result, postpones the transition from selection to integration. Figure 6 shows these effects in Experiment 1.

### Experiment 2

The goal of this experiment is to study the method in the presence of an added unreliable sensor (noise). The new sensor's reading is uniformly distributed noise. In other words, there is no correlation between the position of the stimulus and the sensor's reading. By adding this sensor, the size of the joint state-action space jumps to 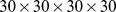.

**Figure 7:**
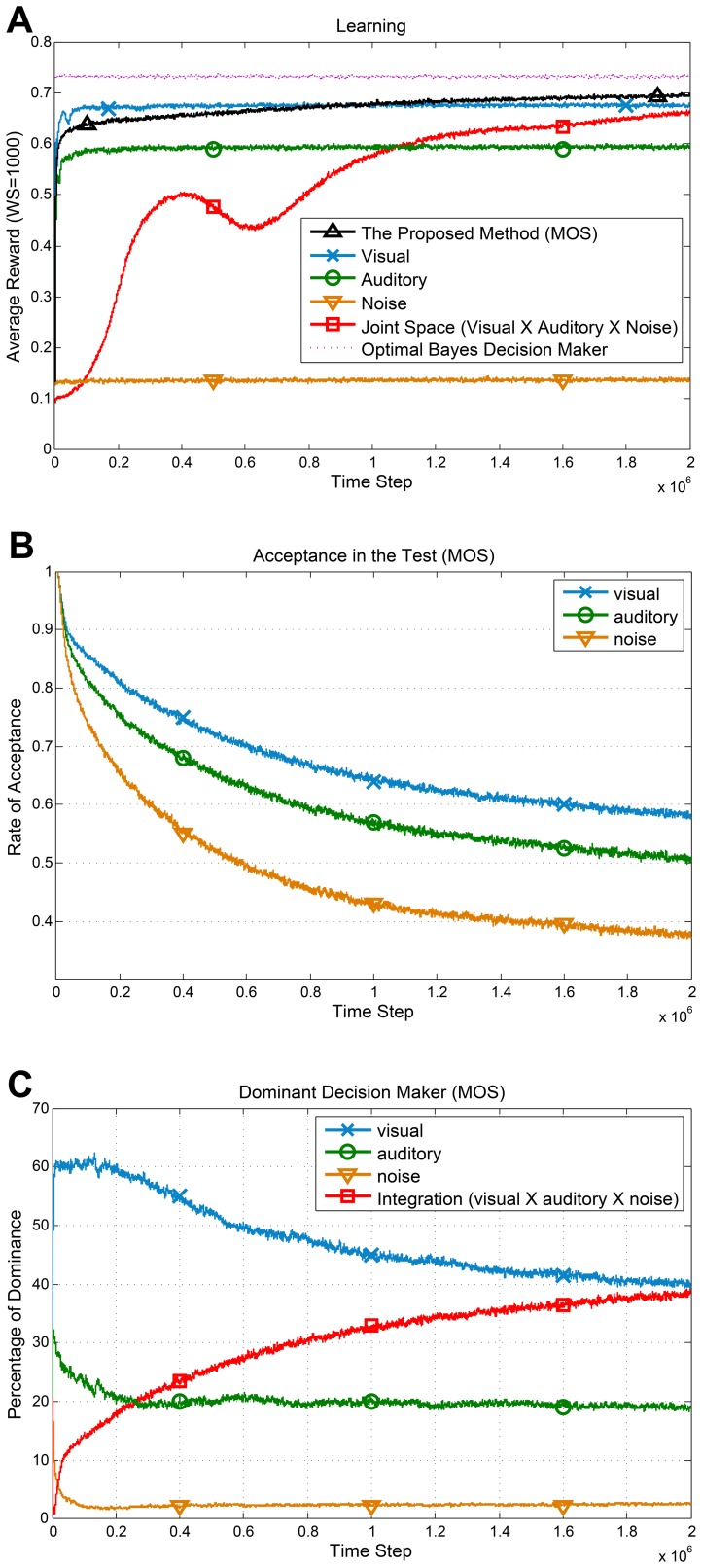
Performance and behavior of the method in response to an unreliable sensor. All graphs are results of averaging over 20 independent runs and passing a moving average window with size 1000. (**A**) Average reward for all agents. For the proposed method (MOS), we used Table 3, employing bound (1) with 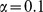 for calculating confidence intervals. The rival methods employ the UCB1 policy on the individual sensors and on the joint space. (**B**) Average acceptance rate (1–rejection rate) of the individual sensors in the proposed method (MOS). (**C**) The average dominancy percentage of each source in decision making (MOS). Due to unreliability of the noise sensor, it takes longer for learning in the integrated states to mature and, therefore, dominancy of the visual sensor is prolonged.

The Noise agent has no beneficial learning and its average reward curve is flat throughout its life; see Figure 7A. Furthermore, due to the presence of this unreliable sensor, learning by the joint space agent has been drastically diminished compared to the Visual agent. The proposed method (MOS) has been able to identify the unreliable source of information and therefore, has been superior to the joint space agent in terms of both learning speed and average reward. However, during the initial steps of learning, its average reward is slightly lower than the Visual agent. It is the cost of having no prior information about the unreliable sensor which makes the method to explore more at the early steps of learning.

The results of the proposed test and the percentage of dominance of each source of information in decision making are shown in Figure 7B and Figure 7C, respectively. The rate of acceptance for all subspaces declines by time and this decline is faster for the unreliable sensor. Moreover, according to Figure 7C, only about 3% of the time the unreliable sensor chooses the final decision. This noise selection mostly contains explorative decisions. This result is evidence that the proposed method clearly considers a subsection of its state space as unreliable and filters it in the decision makings.

#### Comparisons

Table 4 illustrates learning speed in terms of the number of time steps required for each method to reach a certain percentage of the accumulated reward that the Bayesian optimal decision maker achieves. Table 4 also shows the percentage of dominance for each source of information. In all variations of the proposed method, the percentage of dominance for sensory integration increases by progress of learning. Also in the second experiment, the dominance of the noise sensor decreases with time steps. The results indicate that presence of the unreliable sensor in the joint space has made the method slower in the second experiment. This is because the agent has to live with its reliable individual sensors until its joint space yields a reasonable amount of samples to be considered reliable.

We proposed two methods for decision making; namely MOS and LUS, see Table 1 and Table 2. The MOS method chooses the most optimistic source of information, while LUS attends the source with the lowest uncertainty. Both of these criteria are plausible choices for decision making and in our experience both and even some combinations of them work well in practice. Based on Table 4, the LUS method requires fewer time steps compared to the MOS method to reach a certain percentage of performance in both experiments.

#### Confidence intervals

Due to the extreme conservative nature of bounds (2) and (3), for the same 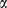, their learning speed is slower than bound (1) in most cases. On the bright side, these bounds are mathematically valid for all kinds of reward distributions. To compensate for this conservativeness, it is recommended to use larger values for 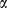 (smaller confidence coefficients) when employing bounds (2) and (3). Furthermore, as mentioned in Method Section, bound (3) is only appropriate in situations where the variances of the reward distributions are small. However, in most cases, there is no information available about the type of the reward distributions and their variances. In these general situations, bound (2) with a moderate value for 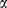 is a reasonable choice. For example, in both of the discussed experiments, by using bound (2) and increasing the value of 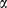 to 0.4, we achieved similar learning speed and average reward to those illustrated in Figure 4A and Figure 7A. A summary of these results is shown in Table 4.

#### Extension to the power set of sensors

**Figure 8:**
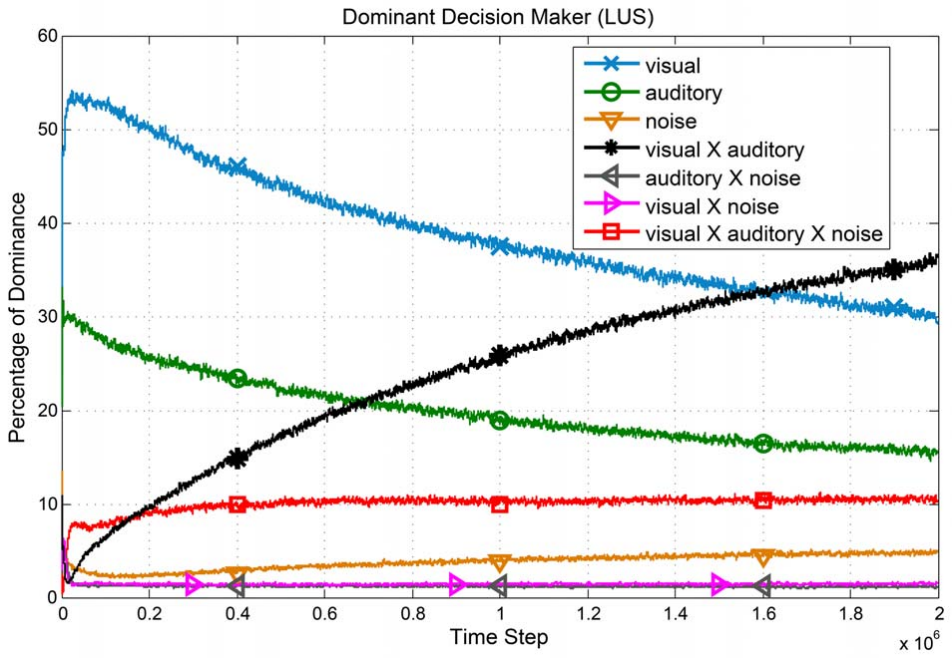
Dominancy of subspaces over time. The average dominancy percentage of different combination of sensors in decision making (LUS). Subspaces including the unreliable source have been filtered. Furthermore, dependency on the integration of reliable sensors increases over time.

Throughout this paper, only individual sensors along with their joint space were considered as the sources of information. However, by a slight modification in equations (4)–(7), we can calculate the necessary marginal values for any combination of sensors. Based on this idea, instead of 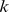 sensors, we can create 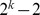 sources of information beside the primary joint space. By employing these sources instead of the individual sensors in line 5 of Table 3, a new variation of the proposed method will be formed. Considering this modification in the algorithm, we performed Experiment 2 with the LUS method using bound (1) and 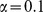. The percentage of dominance of each source of information is shown in Figure 8. In the first section of learning, the final decision is mostly based on the reliable individual sensors and vision is the dominant modality. However, as the agent matures, the most reliable source of information, which is visual
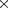
auditory subspace, takes the main role in decision makings. It means that the extended method has the ability to autonomously elicit the reliable subspaces and to filter the unreliable subspaces of its state space. This modification does not change the amount of required memory. However, the new processing complexity will be exponential, which is still reasonable for tasks with a few sensors.

## Discussions and Conclusions

The optimal multisensory integration behavior of adults has been substantially addressed in the literature [1], [2]. However, there are fewer studies and experiments regarding the idea of sensory selection in children [3]–[6]. This lack of sufficient observations is even more significant in the complete age spectral. As a result, there is not sufficient experimental data available to form a definite hypothesis about the transition from sensory selection to sensory integration.

One hypothesis regarding this transition has been proposed by Gori et al. [4], [21]. Their hypothesis is that children select the more accurate sense in multisensory tasks with the purpose of cross-sensory calibration between senses. They suggested that the cross-sensory calibration might have an important impact on maturation of the multisensory perception. In this paper, we have illustrated that even in absence of the cross-sensory calibration hypothesis, the mere transition from the accurate subspaces to the joint space has its own computational advantages. This smooth transition not only facilitates maturation of the multisensory perception, but it is also essential for having a rewarding life.

To show these advantages, we proposed a general multisensory learning method (see Method and Table 3). The proposed method has the ability to autonomously choose different subsets of its state space based on their generalization property and reliability for decision making. Unlike the Bayesian framework, our method neither makes any prior assumptions about the observation model of sensors nor about the relation between sensory space and actions.

It was shown that for an agent who starts its life in a tabula rasa state, the seemingly optimal behavior is to rely on its individual sensors during early life, and to switch to the joint space (sensory integration) in later stages. This behavior is compatible to the empirical findings. Experimental data indicate that children do not integrate sensory information and make their judgments based only on one sensor, whereas adults use multisensory integration for their decision making [3]–[6]. It was also shown that the proposed method is significantly superior to the individual sensor agents (sensory selection alone) and the joint space agent (only sensory integration) in terms of both learning speed and average reward. Based on these findings, we suggest that this selection and integration, which may be interpreted as two separate methods for decision making, are in fact two sides of a coin and both serve the reward maximization behavior. In addition, the transition from selection to integration is a developmental phenomenon and is smooth.

In our framework, the integration-based decisions will become dominant only after the agent receives enough multisensory experiences during the initial stages of its life. There is also similar empirical evidence that the maturation of the integration decisions is related to the early life experiences (see [22], [3]). Moreover, in [10] the authors showed that by using the reward dependent framework, the problem of causal inference in multisensory perception [23] could also be solved in an interactive fashion. For showing this, they used an artificial neural network for calculating the average reward statistics in the joint sensory space. Based on the average rewards, they used a softmax policy for decision making. With some simplifications, we can say that their agent is inherently equivalent to the joint space agent used in our work. The main focus of Weisswange et al. [10] is on the ability of the learning agent to reach the performance of the Bayesian optimal observer. In our work, on the other hand, we have investigated the role of subspace selection in efficiency of interactive learning. Our results justify that our method can reach the performance of the Bayesian optimal observer as well. On top of that, our method justifies the switch from selection to integration in terms of reward maximization. These studies along with our results indicate that by considering the reward dependent framework, we can model (at least in the behavioral aspect) most of the age-related sensory integration phenomena, without making unnecessary mathematical assumptions about the sensor system and the task.

In Experiment 2 it was shown that the algorithm is also plausible in situations where there is a completely unreliable source of information in the joint space. Even in this extreme scenario, our method outperforms its competitors but faces a slight decrease in the learning speed during initial steps. This decrease is indispensable for any interactive learning method which explores different sources of information.

We assumed that the environment is stationary; i.e. the reward distributions are time invariant, or in other words, the sensory models are fixed throughout the learning. These assumptions are widely used in the learning literature. Nevertheless, interactive learning methods can inherently track non-stationary situations; but of course with a lag due to being experienced-based. We discuss this point more in the sequel. In Figure 5 it is shown that the algorithm (using MOS) tracks the sudden change in the environment, called unexpected uncertainty [24], and adapts itself. Nevertheless, there are some methods to directly deal with unexpected uncertainty. For example, a solution is recalculation of the required statistics after detection of an unusual behavior from the environment. This can easily be done by saving the received rewards in a moving window (a short-term memory) and calculating the necessary statistics accordingly [25].

In this work, for simplicity, we used tables for storing the required statistics. This naturally results in the discretization of the state space. Nevertheless, our approach can be generalized to continuous spaces by using the idea of function approximation for estimating the required statistics in Table 3. We believe that to demonstrate the subspace selection behavior of the proposed method for the task at hand, a simple discrete state space is a well-suited balance of complexity and simplicity. However, in our future works we will investigate and test the theory of continuous version of our algorithm in more complex and practical tasks.

In summary, the proposed algorithm is a dynamic subspace selection method for decision making in interactive learning frameworks. Our method intelligently evades the curse of dimensionality problem by exploiting inherent perceptual aliasing in subspaces. This results in fast learning in addition to an efficient and self-governing transition from sensory selection to integration. This transition is essential for having a rewarding life. In addition, the proposed algorithm (Table 3) is easily implementable. These properties make our method an appropriate candidate for lifetime learning of artificial agents having a large number of sensors. Therefore, an important direction of our research team is to extend the current single-step algorithm to a general multi-step learning and decision making algorithm (reinforcement learning). Based on the value-based decision making framework proposed in [9], we can categorize the main contribution of our algorithm in the representation phase where given a set of sensory inputs, the goal is to achieve the most rewarding state representation.
