# Correction: Reward Maximization Justifies the Transition from Sensory Selection at Childhood to Sensory Integration at Adulthood

**DOI:** 10.1371/journal.pone.0115926

**Published:** 2014-12-12

**Authors:** The PLOS ONE Staff

## Notice of Republication

This article was republished on November 3, 2014, to correct errors in equations and in the text of the paper that were introduced during the typesetting process. Please download this article again to view the correct version. The originally published, uncorrected article and the republished, corrected article are provided here for reference.

## Supporting Information

File S1 Originally published, uncorrected article.
(PDF)

File S2 Republished corrected article.
(PDF)
